# Retroversion of Uterus—Retention of Urine for Fifteen Days—Fatal Inflammation of the Bladder

**Published:** 1847-01

**Authors:** 


MANCHESTER PATHOLOGICAL SOCIETY.

*Retroversion of Uterus—Retention of Urine for fifteen days—Fatal Inflammation of the Bladder.*—Mr. Dumville presented the genito-urinary organs of a woman, aged 22, who died in the fourth month of pregnancy from acute inflammation of the bladder consequent on retroversion of the uterus. The body was examined twenty-four hours after death. The bladder contained some urine; its peritoneal covering was thickened and opaque, and had contracted adhesions with the surrounding intestines. There was also some muscular hypertrophy. The whole inner surface was thickly coated with false membrane, which hung in shreds, and was mixed with earthy matters. The uterus was firmly impacted in the pelvic cavity; it contained a four months' fœtus, and the right ovary had within it the corresponding corpus luteum.

The woman had borne two children, and in the latter months of the second pregnancy suffered from prolapsus of the Uterus, which disappeared after delivery. She became a third time pregnant, and during the entire four months preceding death she had suffered from pain in the pubic region, and had also the sensations of prolapse, without, however, any well-ascertained descent of the parts. Twenty-two days previous to death, whilst occupied with manual labour, she experienced a sudden and violent pain in the hypogastrium, with great desire, but inability, to void urine. A surgeon drew off three pints of water, but neglected to repeat the operation for sixteen days. During this interval the bladder was never properly evacuated, the urine mostly escaping guttatim, and with extreme suffering. She was then first visited by Mr. Dumville, who, on examination, discovered a retroversion of the uterus, with the os resting on the symphisis pubis. Marked symptoms of acute inflammation of the bladder presented themselves, which he treated in the usual manner. On introducing the catheter, he withdrew a large quantity of urine, which was strongly ammoniacal, and yielded albumen plentifully. It was of a blood-red colour, and 1012 specific gravity. Microscopically examined, it was found to contain blood-discs, pus globules and crystals of neutral triple phosphate of ammonia and magnesia.

The inflammation of the bladder in this case was, in a certain sense, attributable to the retroversion of the uterus, but still more clearly to the neglect of periodically withdrawing the urine by catheter; for the distension caused by the retained urine, and its irritating properties, determined the fatal issue; so much the more to be regretted, seeing the rules for the management of similar cases have been so distinctly defined that no apology remains for the omission of putting them into practice.—*Dub. Med. Press.*

